# Transcranial Direct Current Stimulation over the Medial Prefrontal Cortex and Left Primary Motor Cortex (mPFC-lPMC) Affects Subjective Beauty but Not Ugliness

**DOI:** 10.3389/fnhum.2015.00654

**Published:** 2015-12-08

**Authors:** Koyo Nakamura, Hideaki Kawabata

**Affiliations:** ^1^Graduate School of Human Relations, Keio UniversityTokyo, Japan; ^2^Department of Psychology, Keio UniversityTokyo, Japan

**Keywords:** neuroaesthetics, esthetic evaluation, tDCS, medial prefrontal cortex, left primary motor cortex

## Abstract

Neuroaesthetics has been searching for the neural bases of the subjective experience of beauty. It has been demonstrated that neural activities in the medial prefrontal cortex (mPFC) and the left primary motor cortex (lPMC) correlate with the subjective experience of beauty. Although beauty and ugliness seem to be semantically and conceptually opposite, it is still unknown whether these two evaluations represent extreme opposites in unitary or bivariate dimensions. In this study, we applied transcranial direct current stimulation (tDCS) to examine whether non-invasive brain stimulation modulates two types of esthetic evaluation; evaluating beauty and ugliness. Participants rated the subjective beauty and ugliness of abstract paintings before and after the application of tDCS. Application of cathodal tDCS over the mPFC with anode electrode over the lPMC, which induced temporal inhibition of neural excitability of the mPFC, led to a decrease in beauty ratings but not ugliness ratings. There were no changes in ratings of both beauty and ugliness when applying anodal tDCS or sham stimulation over the mPFC. Results from our experiment indicate that the mPFC and the lPMC have a causal role in generating the subjective experience of beauty, with beauty and ugliness evaluations constituting two distinct dimensions.

## Introduction

People experience beauty or ugliness in paintings, music, faces, and even mathematical formulae (Aharon et al., 2001; Ishizu and Zeki, 2011; Zeki et al., 2014). Interest has been growing over the past decade about how neural and cognitive systems generate the esthetic experience (Cela-Conde et al., 2004; Kawabata and Zeki, 2004; Leder et al., 2004; Vartanian and Goel, 2004). Several functional magnetic resonance imaging (fMRI) studies have shown that the medial prefrontal cortex (mPFC), including the frontal pole, the dorsal frontomedian cortex, the medial orbitofrontal cortex (mOFC), and the ventromedial prefrontal cortex (vmPFC) are activated when people judged objects to be beautiful (Kawabata and Zeki, 2004; Jacobsen et al., 2006; Ishizu and Zeki, 2011; Jacobs et al., 2012). Interestingly, neural activation in these regions correlates with the experience of beauty derived from various visual categories (e.g., portrait, landscape, still life, or abstract paintings; Kawabata and Zeki, 2004), and modalities (i.e., visual or auditory Ishizu and Zeki, 2011), suggesting that the mPFC responds to beauty beyond its source. Furthermore, there is some evidence for spontaneous evaluation of beautiful objects in the mPFC; in fact, beauty can be assessed even without requiring explicit esthetic evaluation (Kühn and Gallinat, 2012). These findings imply that subjective esthetic evaluation automatically engages a reward system in the brain that is involved in fundamental neural processes such as value-based decision making, preference formation, and choice behavior (Kable and Glimcher, 2009; Grabenhorst and Rolls, 2011).

In contrast, neural activation in the mOFC was weaker when evaluating paintings judged as ugly (Kawabata and Zeki, 2004); activation was increased in the left primary motor cortex (lPMC) and amygdala during the subjective experience of ugliness (Ishizu and Zeki, 2011; Kühn and Gallinat, 2012), the latter of which has been implicated in negative emotion perception (e.g., Armony and Dolan, 2002). Thus, esthetic evaluation can engage the reciprocal interaction of neural activities from areas including the mPFC and the lPMC. In most previous neuroaesthetic studies, participants were asked to evaluate how beautiful the presented artworks were on a scale ranging from ugly to beautiful in a unitary dimension (e.g., Kawabata and Zeki, 2004; Ishizu and Zeki, 2011). However, whether beauty and ugliness represent extreme opposites in a unitary dimension remained controversial in philosophical and psychological fields (Cacioppo and Berntson, 1994; McConnell, 2008). We thus wondered whether evaluating beauty is simply the opposite of evaluating ugliness, and whether these evaluation share common neural substrates. Accumulating psychological and neurophysiological evidence has demonstrated that positive and negative (e.g., likability and dislikability, attractiveness, and unattractiveness) evaluations are represented independently as two dimensions in the brain (Fiorillo, 2013; Jessup and O’Doherty, 2014). For example, dopamine neurons in a rhesus macaque that selectively encode a reward value are insensitive to aversion (Fiorillo, 2013). Likewise, the mPFC is uniquely involved in encoding gain outcomes, while the bilateral OFC selectively encodes loss outcomes (Jessup and O’Doherty, 2014). In addition, the subjective experiences of negative emotion and ugliness partially share neural substrates, and especially activate the right inferior frontal gyrus (IFG; Yeh et al., 2015). Given this functional distinction between positive and negative evaluations, it is therefore possible that the neural processing necessary for the evaluation of beauty and ugliness is dissociable, and that the reward circuit in the human brain is engaged critically when people evaluate how beautiful an object is, but not how ugly it is, although these evaluations are seemingly opposites in esthetic evaluation. Given the previous evidence, people can see both beauty and ugliness in the same object at the same time, engaging distinct neural systems.

Although neuroaesthetics has identified the neural correlates of beauty and ugliness, to the best our knowledge, no study has directly investigated how evaluating beauty and ugliness are qualitatively different and whether these evaluations constitute two distinct dimensions. Understanding how people judge things as ugly is essential for a complete representation of esthetic evaluation—do subjective beauty and ugliness arise from the same cognitive and neural mechanism? Furthermore, although neuroimaging studies repeatedly observed that evaluating beauty engages the mPFC and the lPMC, they are primarily correlational and therefore poorly suited for demonstrating causality. To further understand the role of the mPFC and the lPMC on the two types of esthetic evaluation, it is important to pursue the causal role of the mPFC and the lPMC in the appreciation of beauty and ugliness. One possible method is to employ non-invasive brain stimulation methods such as transcranial direct current stimulation (tDCS), which produces temporal virtual lesions or enhancements to specific parts of the brain. tDCS modulates neuron’s resting membrane potential by passing a low-intensity current from an anode to a cathode on the scalp (Filmer et al., 2014). In general, anodal stimulation inhibits neurotransmission through γ-aminobutyric acid (an inhibitory neurotransmitter), which induces depolarization of the resting membrane potential, eventually leading to the excitability of neural activities in the area where stimulation is applied. Meanwhile, cathodal stimulation inhibits neurotransmission by glutamate (an excitatory neurotransmitter), which causes hyperpolarization of the resting membrane potential, leading to decreases in neural activity (Nitsche and Paulus, 2000; Filmer et al., 2014). Recent studies with tDCS suggested that brain stimulation could be used to investigate brain mechanisms underlying reward-related behavior (for a review, see Levasseur-Moreau and Fecteau, 2012). Specifically, subjective evaluation can be modulated by the electrophysiological state of the dorsolateral prefrontal cortex (DLPFC; e.g., Cattaneo et al., 2013; Chib et al., 2013), known to play a critical role in top-down modulation in decision making (Hare et al., 2009), even though it is elicited artificially.

The main aim of this study was thus to investigate the causal relationship between neural activity in the mPFC and the lPMC, and subjective evaluation of beauty and ugliness, through the application of tDCS over the mPFC and the lPMC, which are known to be closely related to the evaluation of beauty. We also examined how this neural modulation would affect the perception of beauty and ugliness in paintings. If evaluation of beauty and ugliness were neurally represented as extreme opposites in a unitary dimension, then tDCS over the mPFC and the lPMC should modulate the evaluation of beauty and ugliness equally. However, with the functional distinction between positive and negative evaluation in mind (Cacioppo and Berntson, 1994), we hypothesized that tDCS over the mPFC and the lPMC would be effective in inducing changes in the evaluation of beauty but not for ugliness. We used both self-reported evaluations (measured by explicit rating) and automatic esthetic evaluation (measured by response latency in esthetic judgment).

## Materials and Methods

### Participants

Forty-seven right-handed Japanese students (30 women, mean age = 21.02 years, *SD* = 1.25, range = 19–24 years) participated in this study. Sample size was determined on the basis of a previous study on esthetic judgment with a between-subjects design using tDCS (Chib et al., 2013), which recruited on average 16.5 participants per stimulation group (range: 14–20 participants). The data collection stop rule was to enroll at least eligible 14 participants per stimulation group and to stop at 20 participants per group. All had normal or corrected-to-normal vision, and none had a history of neurological or psychiatric disorder. Participants gave written informed consent before participating, and the study was approved by the local ethical committee of Keio University, Japan. All procedures were carried out in accordance with the Declaration of Helsinki. Two female participants in the sham condition were excluded from data analysis because their mean beauty rating scores before brain stimulation were extremely low (exceeded -2.5 SD).

### Apparatus and Stimuli

The stimuli were 90 20th century abstract paintings by 90 different painters obtained from a web gallery, *Wikipaintings* (http://www.wikipaintings.org/). In our preliminary experiment, a separate group of five participants (two women; mean age = 21.80 years, *SD* = 1.79, range = 19–24 years) scored the perceived beauty of a set of 180 paintings on a scale ranging from 1 (*not beautiful at all*) to 9 (*extremely beautiful*). According to these participants’ mean rating scores, 90 paintings that were scored as moderately beautiful were selected from the stimulus set (*M* = 4.60, *SD* = 0.49), and finally used as the experimental stimuli in the main study. All stimuli were adjusted to the same height (approximately 15° in visual angle), and were presented on a 21-inch CRT monitor (Trinitron CPD-G420, SONY) with a refresh rate of 100 Hz and a screen resolution of 1280 pixels × 960 pixels. The experiment was controlled by MATLAB (MathWorks, Natick, MA, USA) using a MacBook Pro (MacBook Pro, Apple, USA). Participants sat at a viewing distance of 57 cm away from the monitor with a chinrest.

### Procedure

#### Pre-/Post-stimulation Sessions

The main experiment was divided into three parts: a pre-stimulation session, a tDCS session, and a post-stimulation session. Before the tDCS session, participants were asked to complete all the following tasks: (i) beauty rating task, (ii) ugliness rating task, and (iii) beauty-ugliness judgment task. Note that the post-stimulation session was initiated within 1 min from the end of the tDCS session. In this session, participants were required to complete the same tasks as in the pre-stimulation session.

In the beauty rating task, participants were required to rate the subjective beauty of each painting on a scale ranging from 1 (*not beautiful at all*) to 9 (*extremely beautiful*), using a visual slider scale controlled by a mouse. Each trial started with presenting a fixation cross at the center of the screen for 500 ms followed by the presentation of a painting stimulus with the visual slider scale. The rating score for the presented painting was determined by a mouse click. Participants were able to view the painting until their response was made. Each painting stimulus was presented once in random order.

In the ugliness rating task, participants were asked to rate the subjective ugliness of each painting on a scale ranging from 1 (*not ugly at all*) to 9 (*extremely ugly*), identical to the beauty rating task. During both the beauty and ugliness rating tasks, participants were reminded that the ratings were subjective and encouraged to use the entire scale across all the paintings. Prior to each rating task, subjects completed a short practice session using the paintings that were not used in the rating tasks. The order of these two tasks was counterbalanced across participants.

After completing the rating tasks, the beauty-ugliness judgment task was conducted. This task was designed to measure the time it takes to judge the beauty or ugliness of the paintings. Previous studies demonstrated that positive evaluation (i.e., likability) could be relatively rapid compared to negative evaluation because positive evaluation is made without reference to negative evaluation, while negative evaluation is made under the reference of positive evaluation (Herr and Page, 2004; Herr et al., 2012). In this task, each of the 90 paintings was paired with the Japanese character that indicates either “beauty” or “ugliness,” making 180 trials in total. Participants were required to judge whether their own esthetic reaction to the painting (i.e., beautiful or ugly) was consistent with the meaning of the character. On each trial, a painting was presented on the screen for 750 ms, and then replaced by the character (i.e., the character indicating beauty or ugliness). If participants judged the character as consistent with their own esthetic reaction to the painting, then they were to press the key corresponding to “consistent” as quickly and accurately as possible. If participants judged that the description did not fit their own esthetic reaction, then they were to press the key labeled “inconsistent.” All key presses were made on the right hand side. All paintings were presented in randomized order. Prior to this task, subjects completed 20 practice trials, with abstract paintings different from the ones used in the main task, to familiarize themselves with the task. The order of the tasks in the post-stimulation session was conducted in the same order as in the pre-stimulation session.

In the pre-stimulation session, mean task durations were 7.25 ± 2.11 min for the beauty rating task, 6.98 ± 1.60 for the ugliness rating task, and 8.33 ± 0.14 for the beauty-ugliness judgment task. In the post-stimulation session, mean task durations were 6.34 ± 1.23 min for the beauty rating task, 6.45 ± 1.45 min for the ugliness rating task, and 8.34 ± 0.17 min for the beauty-ugliness judgment task.

#### Transcranial Direct Current Stimulation (tDCS)

Immediately after the pre-stimulation session, participants were stimulated with tDCS for approximately 15 min (the tDCS session). During stimulation, participants were required to remain seated and relaxed. In this session, participants spent the time listening to classical music. The tDCS was applied using a battery-driven DC stimulator (DC-STIMULATOR PLUS, NeuroConn, Ilmenau, Germany) through a pair of saline-soaked sponge electrodes (5 cm × 5 cm, 25 cm^2^). The stimulation areas of interest (the mPFC and lPMC) were determined using the standard 10–20 electroencephalography (EEG) system and anatomical landmarks. To stimulate the mPFC, the electrode was placed on the frontal pole with reference to halfway between Fp1 and Fp2 and the glabella. The lPMC was targeted with reference to C3. Participants were blinded for stimulation condition (single-blinded design) and randomly assigned to one of three groups: mPFC anodal, mPFC cathodal, and sham. None of participants had any previous knowledge of tDCS and any experience of stimulation, and were aware of type of stimulation they received, whereas the experimenter was fully informed. Participants in the mPFC anodal group (*n* = 15) received active stimulation in the mPFC with the cathodal electrode over the lPMC, while participants in the mPFC cathodal group (*n* = 15) received active stimulation in the mPFC with the cathodal electrode. Participants in the sham group received sham stimulation over the same cortical areas as the PFC anodal (*n* = 7) and mPFC cathodal groups (*n* = 8), respectively. Participants in the three groups did not differ in sex [χ^2^(2) = 0.19, *p* = 0.91] or age [*F*(2,42) = 1.02, *p* = 0.37]. For the mPFC anodal and the mPFC cathodal groups, stimulation intensity was set at 2 mA and the duration of stimulation was approximately 15 min. We ramped the current up over the first 40 s of the stimulation and down over the last 40 s. It has been demonstrated that 20 min of 2 mA anodal stimulation results in excitability enhancement that is still observable 90 min after the end of the stimulation (e.g., Batsikadze et al., 2013). For the sham condition, the stimulator was turned on for only 15 s. Thus, participants in the sham group felt the initial itching induced by the tDCS but received no stimulation for the rest of the stimulation period. A post-stimulation self-report questionnaire (rated on a scale of 1–9, with 9 being the most uncomfortable) confirmed that the discomfort associated with itching induced by the tDCS did not differ between the stimulation conditions [*F*(2,42) = 0.55, *p* = 0.58].

## Results

### Change in the Experience of Beauty

**FIGURE 1:**
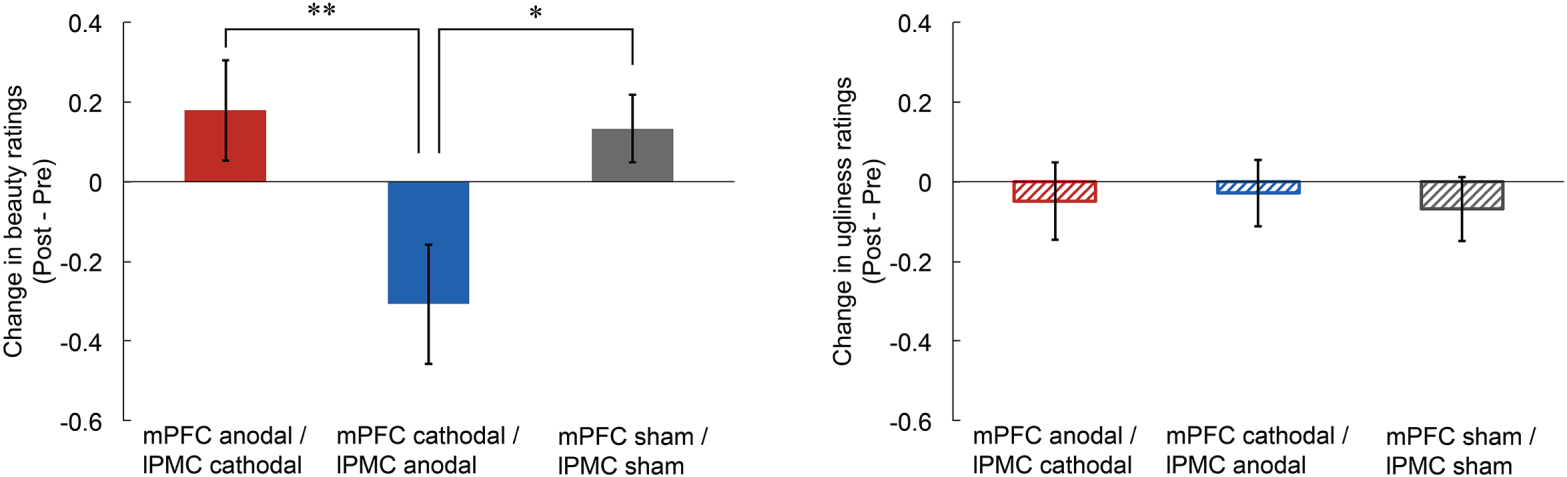
**Post-stimulation change in rating scores of beauty and ugliness.** The beauty rating in the mPFC cathodal significantly decreased compared to those in the sham and the mPFC anodal **(left panel)**. Anodal or cathodal stimulation of the mPFC did not change ugliness rating score **(right panel)**. Error bars represent standard errors (SEs) of the mean. Asterisks indicate the results of multiple comparisons (^∗^*p* < 0.05; ^∗∗^*p* < 0.01).

To investigate the effect of tDCS over the mPFC and the lPMC on the experience of beauty, we first calculated any change in beauty ratings by subtracting rating scores in the pre-stimulation from those in the post-stimulation for each participant (left panel of **Figure 1**). Then, we conducted an ANCOVA on the change in beauty ratings with group as the between factor (mPFC anodal vs. mPFC cathodal vs. sham) and with the pre-stimulation rating scores as covariate so that individual differences in the pre-stimulation session could be statistically controlled. The results showed a significant main effect of group [*F*(2,41) = 4.64, *p* < 0.05, $\eta_{p}^{2}$ = 0.18], but there was no significant main effect of pre-stimulation rating scores [*F*(1,41) = 0.08, *p* = 0.78, $\eta_{p}^{2}$ = 0.00]. A one-way ANOVA without the use of pre-stimulation rating as covariate was also significant [*F*(2,42) = 4.74, *p* < 0.05, $\eta_{p}^{2}$ = 0.18]. Subsequent multiple comparisons (Ryan’s method) revealed that the beauty rating in the mPFC cathodal significantly decreased compared to those in the sham [*t*(42) = 2.52, *p* < 0.05, *r* = 0.36] and the mPFC anodal [*t*(42) = 2.79, *p* < 0.01, *r* = 0.40]. Supplemental analysis confirmed that the rating scores in the three stimulation groups did not differ in the pre-stimulation session (see Supplementary Figure S1), and tDCS did not affect response latency (see Supplementary Figure S2).

### Change in the Experience of Ugliness

To examine the effect of tDCS on the experience of ugliness, we first calculated any change in ugliness ratings by subtracting rating scores in the pre-stimulation from those in the post-stimulation for each participant. Then, we conducted an ANCOVA on change in ugliness ratings with group as the between factor (mPFC anodal vs. mPFC cathodal vs. sham) and with the pre-stimulation rating scores as covariate. The results showed no significant main effect of group [*F*(2,41) = 0.05, *p* = 0.95, $\eta_{p}^{2}$ = 0.00] or of pre-stimulation rating scores [*F*(1,41) = 0.14, *p* = 0.71, $\eta_{p}^{2}$ = 0.00]. These results indicate that none of the tDCS applied in this study affected the experience of ugliness (right panel of **Figure 1**; see also Supplementary Figure S6). Supplemental analysis confirmed that rating scores in the three stimulation groups did not differ in pre-stimulation session (see Supplementary Figure S3), and tDCS did not affect response latency (see Supplementary Figure S4).

### Correlation between Ratings of Beauty and Ugliness

We calculated the mean beauty and ugliness rating scores for each painting, creating 90 beauty and 90 ugliness rating scores. Correlation coefficients between beauty and ugliness rating scores were calculated for each group and each session. All correlation coefficients between ratings of beauty and ugliness were statistically significant (*p*s < 0.005), suggesting that beauty and ugliness are partially interchangeable. Further, there were no significant differences between these correlation coefficients [χ^2^(5) = 1.41, *p* = 0.92], and the estimated ρ calculated from these correlation coefficients was -0.48 with a 95% confidence interval of -0.64 to -0.29.

### Response Latencies for Judging Beauty and Ugliness

**FIGURE 2:**
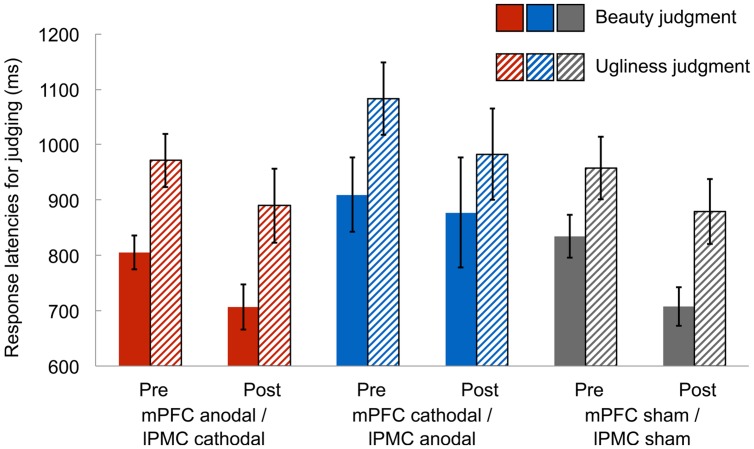
**Mean response latencies for judging beauty and ugliness in the pre-stimulation and post-stimulation session.** In both the post-stimulation and the pre-stimulation sessions, the mean response latencies for judging beauty were shorter than those for judging ugliness in all groups, irrespective of the type of tDCS. Error bars represent standard errors (SEs) of the mean.

In the beauty-ugliness judgment task, we measured the response latencies of judging beauty and ugliness in the paintings to identify any differences in these judgment processes. Mean response latencies were calculated after removing response latencies shorter and longer than 3 SDs below and above the mean in each condition, respectively (1.9% of all data). A 3 (group: mPFC anodal vs. mPFC cathodal vs. sham) × 2 (session: pre-stimulation vs. post-stimulation) × 2 (judgment: beauty vs. ugliness) ANOVA on response latencies revealed a significant main effect of session [*F*(1,42) = 10.23, *p* < 0.005, $\eta_{p}^{2}$ = 0.20], suggesting that response latencies in the post-stimulation session were faster than those in the pre-stimulation session. However, this might simply reflect a learning effect in judging the beauty and ugliness of the paintings due to repeated judgments. Further, there was a significant main effect of judgment [*F*(1,42) = 85.97, *p* < 0.001, $\eta_{p}^{2}$ = 0.67], indicating that participants judged beauty faster than ugliness (**Figure 2**). There were, however, neither significant main effect of group [*F*(2,42) = 1.53, *p* = 0.23, $\eta_{p}^{2}$ = 0.07] nor significant two-way interactions [group × session: *F*(2,42) = 0.15, *p* = 0.86, $\eta_{p}^{2}$ = 0.01; group × judgment: *F*(2,42) = 0.42, *p* = 0.66, $\eta_{p}^{2}$ = 0.02; session × judgment: *F*(1,42) = 0.00, *p* = 0.97, $\eta_{p}^{2}$ = 0.00]. Moreover, there was no significant three-way interaction [*F*(2,42) = 1.88, *p* = 0.16, $\eta_{p}^{2}$ = 0.08]. Supplemental analysis showed that differences in beauty and ugliness judgment were seen in only response latency, and the rate of judging “consistent” did not differ between beauty and ugliness judgment (see Supplementary Figure S5).

## Discussion

This study applied tDCS to examine whether non-invasive brain stimulation modulates esthetic evaluation, and we demonstrated that stimulation over the frontal pole of the prefrontal cortex (likely affecting the mPFC) and the lPMC changed the subjective experience of beauty. Thus, our results extended the findings of previous neuroimaging studies showing the central role of these regions on the appraisal of beauty (Kawabata and Zeki, 2004; Ishizu and Zeki, 2011). Specifically, inhibiting neural excitability in the mPFC by applying cathodal tDCS with anodal tDCS over the lPMC diminished the experience of beauty. On the other hand, enhancing neural excitability in the mPFC by applying anodal tDCS over the mPFC did not significantly influence the perception of beauty. These results indicate that artificially modulating the neural excitability of the mPFC and the lPMC, which is implicated in the evaluation of beauty, can change the subjective experience of beauty.

With respect to the evaluation of ugliness, no differences in ratings were found after applying tDCS over the mPFC and the lPMC, regardless of type of stimulation. This suggests that electrode alignment modulated the evaluation of beauty, but not for ugliness, even though the evaluation of beauty and ugliness has often been conceptualized as being bipolar. Our results are consistent with previous findings that positive and negative evaluations are represented as independent dimensions in the brain (Fiorillo, 2013; Jessup and O’Doherty, 2014; Martín-Loeches et al., 2014). Neuroimaging studies have repeatedly demonstrated that the mOFC is specialized for encoding reward value while the lateral OFC or the IFG respond to the magnitude of punishment, loss, or unpleasantness (Grabenhorst and Rolls, 2011; Yeh et al., 2015). Inferred from the neuroimaging evidence, the evaluation of beauty and ugliness of artworks are potentially distinct dimensions that engage different neural substrates, and the evaluative processes underlying beauty and ugliness are partially interchangeable and reciprocally activated. This was supported by our results that the rating scores of beauty and ugliness were moderately correlated (estimated ρ = -0.48). Nevertheless, the assumption that beauty and ugliness are endpoints on a bipolar scale is not sufficient to explain our results comprehensively, similar to recent behavioral evidence (e.g., Shimojo et al., 2003; Nakamura and Kawabata, 2013). The neural processing underlying the explicit evaluations of beauty and ugliness for the same objects, constituting two relatively distinct evaluative dimensions, should be tested further in the future study.

This functional distinction of beauty and ugliness evaluations was also indicated in response latency for judging beauty and ugliness. In the beauty-ugliness judgment task, participants judged whether the painting was beautiful faster than they did about whether it was ugly. Although the general speed of responding became faster in the post-stimulation session due to the learning effect, the relative difference in response latencies of beauty and ugliness remained the same throughout the experiment. This implies that beauty is automatically evaluated without requiring cognitive effort compared to the evaluation of ugliness. Supplemental results showed that response rates of judging “consistent” did not differ between beauty and ugliness judgment so that difference in response latency of beauty and ugliness judgment should be derived from cognitive processing underlying these evaluations. The beauty for abstract artworks is judged automatically based on instant affective reactions without extended thoughts or intentions (Pavlovic and Markovic, 2012). The relative automaticity of beauty judgment observed in this study is consistent with the explanation by Herr and Page (2004) that people are more disposed to evaluate objects based on its likability rather than dislikability, such that likability is evaluated without reference to dislikability while reporting dislikability necessitates a reference to likability. Along with the authors’ assumption, our findings indicate that the evaluation of beauty is more dominant and fluent compared to that of ugliness, rather than evaluating beauty is completely opposite of evaluating ugliness. It is possible that beauty is evaluated solely without reference to ugliness, while evaluating ugliness requires to same extent the appraisal of how beautiful the object is, whereby beauty and ugliness evaluations are asymmetrically linked in evaluative processes (Herr and Page, 2004; Herr et al., 2012). Thus, our results suggest that cognitive structures underlying beauty and ugliness judgment seem to be separable to some extent. Although there is no direct previous evidence of separability of evaluating beauty and ugliness, studies on positive and negative emotional evaluations are helpful for interpreting our observations. From an evolutionary perspective, framing events, or environments in terms of the outcome people hope to find or approach rather than the logically equivalent absence of the outcome they want to avoid facilitates the motivation to approach and explore novel objects (Herr and Page, 2004). Accordingly, people may tend to predominantly evaluate how beautiful, pleasant, and approachable it is, compared to how ugly, unpleasant, and avoidable it is. This prioritized beauty judgment was robust considering that it was unaffected by any type of tDCS over the mPFC and lPMC. Thus, the effect of tDCS was evident in self-reported evaluation of beauty while brain stimulation did not impact the relative dominance of beauty judgment.

Although our finding that artificially modulating neural excitability elicited a change in esthetic evaluation converges well with earlier studies showing that the mPFC and the lPMC have central roles in esthetic evaluation (Kawabata and Zeki, 2004; Di Dio et al., 2007; Ishizu and Zeki, 2013), we need to consider that the spatial resolution of tDCS using large sponge pads positioned on the skull is relatively diffuse. Given this nature, brain stimulation is unlikely to be constrained to the cortex underneath an electrode (Datta et al., 2009; Bikson et al., 2013; Bestmann et al., 2015; Shin et al., 2015). Recent efforts have been directed toward estimating brain current flow patterns during stimulation by computational modeling of tDCS (Bikson et al., 2012). For instance, Datta et al. (2009) revealed that anodal tDCS over the lPMC with the cathodal electrode over the contralateral orbita using large rectangular pads (7 cm × 5 cm) results in diffuse cortical modulation, based on the highly detailed anatomical computational model. In a similar vein, Manuel et al. (2014) confirmed by a computational modeling that neural excitability of posterior OFC was successfully modulated by anodal tDCS over the frontal pole with the cathodal over the vertex. However, their modeling also indicated that the stimulation targeted on OFC induced widespread frontal modulation, including the DLPFC. Drawing on these findings, it is likely that mPFC stimulation in our experiment actually stimulated the broader areas in frontal region, including the frontal pole, the mOFC, the vmPFC, and DLPFC. Taking this into account, it raises the possibility that stimulation of the mPFC and lPMC led to neural modulation of another frontal region, such as the left DLPFC. This possibility is consistent with the finding of Cattaneo et al. (2013) that anodal tDCS over the left DLPFC leads to changes in beauty evaluation. Considering the complexity of frontal cortex functions, further studies are therefore needed to fully delineate the combination of electrodes for mechanistic understanding of the effect of tDCS on esthetic evaluation. It is conceivable that the specific combination of anodal and cathodal stimulation were required to induce the change in beauty perception, because the different combinations of electrode placements resulted in distinct electric fields induced by tDCS. tDCS provides complex dose-specific changes in brain function (e.g., combinations of electrode placements, intensity, stimulation duration, and so forth). Although the current study applied tDCS and measured behavioral changes, measuring brain activity by applying fMRI or EEG, can reveal the more detailed neural mechanisms underlying subjective evaluation of beauty and ugliness (Saiote et al., 2013).

Another limitation lies in the extent to which the tDCS applied in this study can be generalized to other types of stimuli. We used abstract paintings as stimuli, which lacked obvious realistic contents. The subjective beauty in abstract works could be perceived in both bottom-up and top-down ways. People may perceive the beauty of abstract artworks in a particular set of visual features such as shapes, colors, and texture, while their appraisal of beauty depends on personality and past experience as well (Melcher and Bacci, 2013). In the case of esthetic evaluation of representational artworks, the role of top-down cognitive processing exerted in frontal regions has been emphasized. The left DLPFC is considered to integrate the information required to judge beauty and to exert top-down control in orienting esthetic viewing (Cupchik et al., 2009), leading to a more critical role in the appraisal of representational compared to abstract paintings (Lengger et al., 2007). Consistent with these studies, Cattaneo et al. (2013) found that anodal stimulation on the left DLPFC enhanced beauty experience for representational images, but not for abstract ones. Thus, our results indicate that brain stimulation can influence the experience of beauty on several levels. Further studies will be required to systematically examine the effect of tDCS on the esthetic evaluation of a variety of objects (e.g., faces, music, and odors).

## Conclusion

We found that the subjective experience of beauty but not ugliness can be artificially modulated by tDCS over the mPFC and lPMC, thus providing a new insight that beauty and ugliness judgments constitute two distinct dimensions that engage different neural substrates. This finding contributes to future research by revealing that the evaluation of beauty is not the mere opposite of the evaluation of ugliness, and that these two evaluations should be investigated separately to profoundly understand esthetic evaluation. This functional distinction of beauty and ugliness was also reflected in automatic esthetic evaluation. Extending previous findings that the mPFC and the lPMC plays a central role in the appreciation of beauty, we showed that the mPFC and the lPMC has a causal role in the subjective experience of beauty.

## Conflict of Interest Statement

The authors declare that the research was conducted in the absence of any commercial or financial relationships that could be construed as a potential conflict of interest.
