# Supplementary material for: Transcranial Direct Current Stimulation over the Medial Prefrontal Cortex and Left Primary Motor Cortex (mPFC-lPMC) Affects Subjective Beauty but Not Ugliness
Source: Front Hum Neurosci. 2015 Dec 8;9:654. doi: 10.3389/fnhum.2015.00654 (PMC4672048; doi:10.3389/fnhum.2015.00654)
Supplement: Supplementary file 1 [file Data_Sheet_1.DOCX]

Supplementary Material

Transcranial direct current stimulation over the medial prefrontal cortex and the left primary motor cortex (mPFC-lPMC) affects subjective beauty but not ugliness

Koyo Nakamura^1^, Hideaki Kawabata^2^*

^1^Graduate School of Human Relations, Keio University, Mita, Minato-ku, Tokyo, Japan

^2^Department of Psychology, Keio University, Mita, Minato-ku, Tokyo, Japan

*** Correspondence:**

Dr. Hideaki Kawabata

Department of Psychology, Keio University, 2-15-45, Mita, Minato-ku, Tokyo, 108-8345, Japan.

e-mail: kawabata@flet.keio.jp

# Supplemental results

## Pre- and post-stimulation beauty rating scores in different stimulation conditions

Figure S1 shows the mean beauty rating scores in pre- and post-stimulation sessions. A 3 (group: mPFC anodal vs. mPFC cathodal vs. sham) × 2 (session: pre-stimulation vs. post-stimulation) ANOVA on the mean beauty rating scores revealed a significant interaction effect between group and session (*F*(2, 42) = 4.74, *p* < .05, η_p_^2^ = .18). There were neither significant main effect of group (*F*(2, 42) = 1.14, *p* = .33, η_p_^2^ = .05) nor significant main effect of session (*F*(1, 42) = 0.00, *p* = .98, η_p_^2^ = .00). An analysis of simple main effects revealed a significant main effect of session in the mPFC cathodal group (*F*(1, 42) = 6.21, *p* < .05, η_p_^2^ = .13), indicating that cathodal stimulation of the mPFC led to decrement in beauty rating score. However, there was no significant main effect of session in the mPFC anodal (*F*(1, 42) = 2.11, *p* = .15, η_p_^2^ = .04) and sham group (*F*(1, 42) = 1.16, *p* = .29, η_p_^2^ = .03). Further, a main effect of group in the pre-session was not significant (*F*(2, 84) = 0.81, *p* = .45, η_p_^2^ = .02), confirming that the mean beauty rating scores did not differ between stimulation groups in the pre-stimulation session. For the post-stimulation session, a main effect of group was marginally significant (*F*(2, 84) = 2.45, *p* = .09, η_p_^2^ = .06), in which the rating score in the mPFC cathodal tended to be lower than that of the sham group.


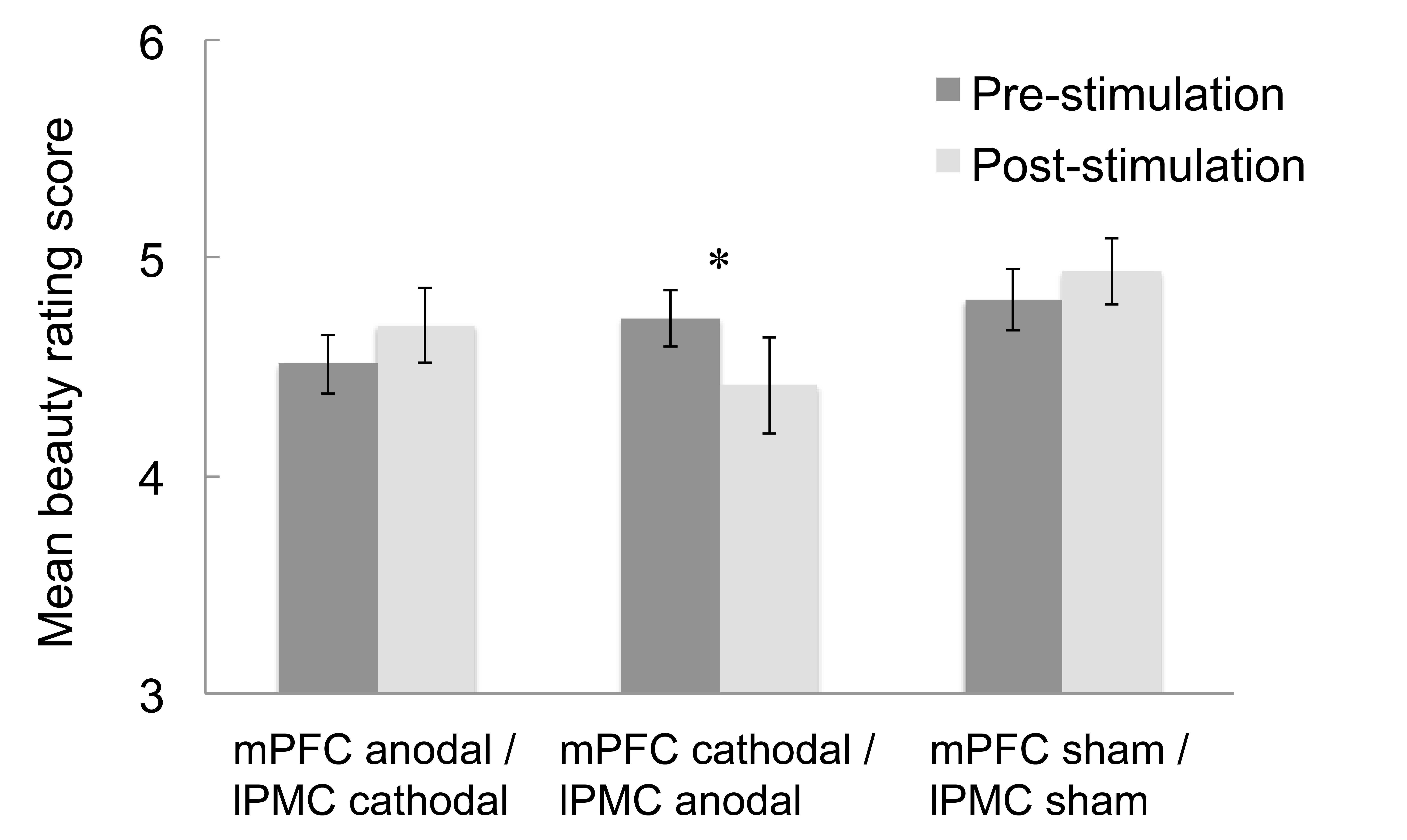


**Figure S1. Pre- and post-stimulation beauty rating scores in different stimulation conditions.** Error bars represent SEs of the mean. Asterisk indicates the result of simple main effects analyses (* *p* < .05).

## Pre- and post-stimulation beauty rating latencies in different stimulation conditions

Figure S2 shows the mean beauty rating latencies in pre- and post-stimulation session. A 3 (group: mPFC anodal vs. mPFC cathodal vs. sham) × 2 (session: pre-stimulation vs. post-stimulation) ANOVA on the mean beauty rating latencies revealed a significant main effect of session (*F*(1, 42) = 17.70, *p* < .001, η_p_^2^ = .30), suggesting that rating latencies in the post-session were faster than those in the pre-session. However, there were neither significant main effect of group (*F*(2, 42) = 0.54, *p* = .59, η_p_^2^ = .02) nor significant interaction effect between group and session (*F*(2, 42) = 0.22, *p* = .81, η_p_^2^ = .01), indicating that tDCS did not affect beauty rating latency.


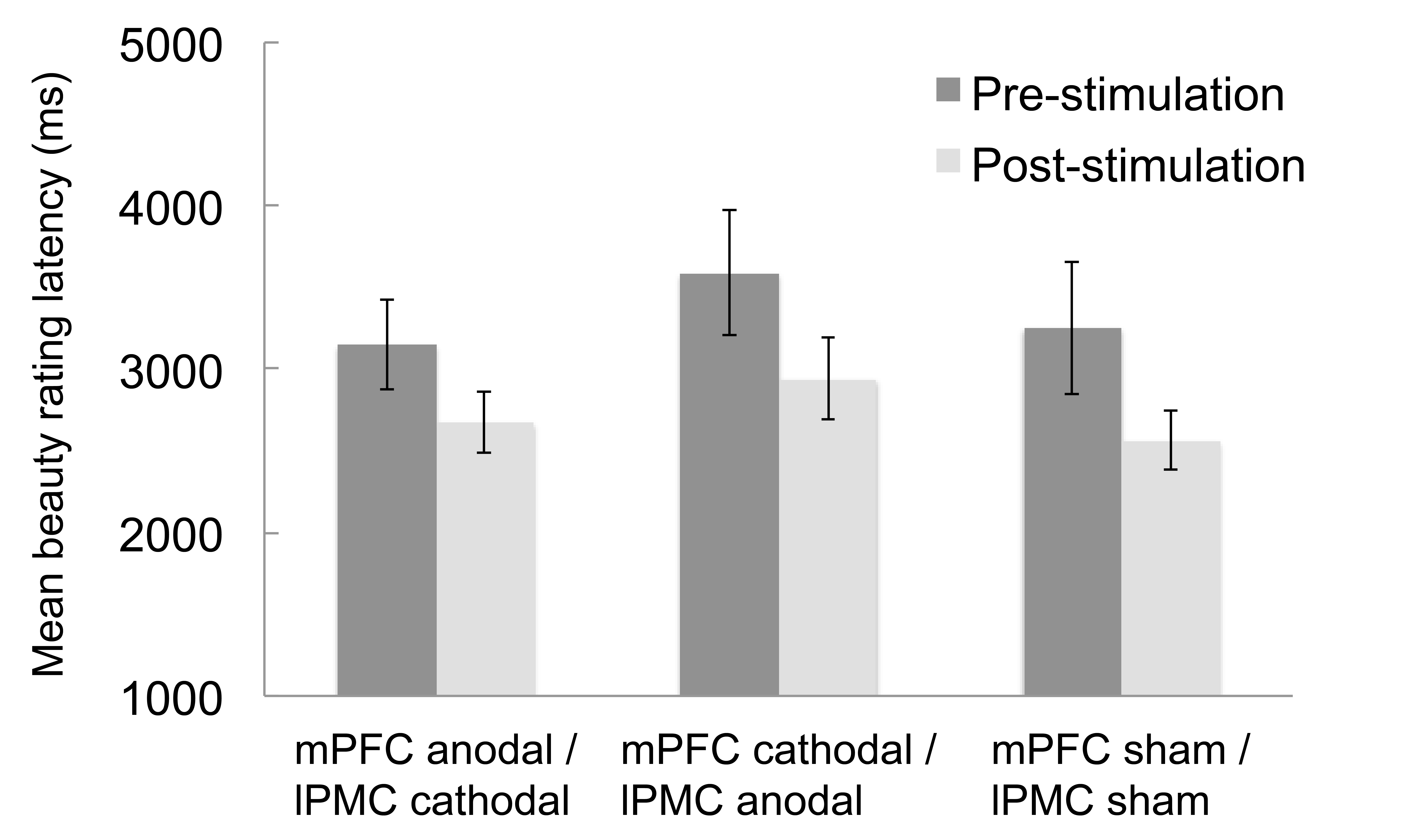


**Figure S2. Pre- and post-stimulation beauty rating latencies in different stimulation conditions.** Error bars represent SEs of the mean.

## Pre- and post-stimulation ugliness rating scores in different stimulation conditions

Figure S3 shows the mean ugliness rating scores in the pre- and post-stimulation sessions. A 3 (group: mPFC anodal vs. mPFC cathodal vs. sham) × 2 (session: pre-stimulation vs. post-stimulation) ANOVA on the mean ugliness rating scores revealed neither significant main effect of group (*F*(2, 42) = 1.61, *p* = .21, η_p_^2^ = .07) nor session (*F*(1, 42) = 0.91, *p* = .34, η_p_^2^ = .02). Further, there were no significant interaction effect between group and session (*F*(2, 42) = 0.05, *p* = .94, η_p_^2^ = .00), suggesting that ugliness rating scores were not changed by tDCS.


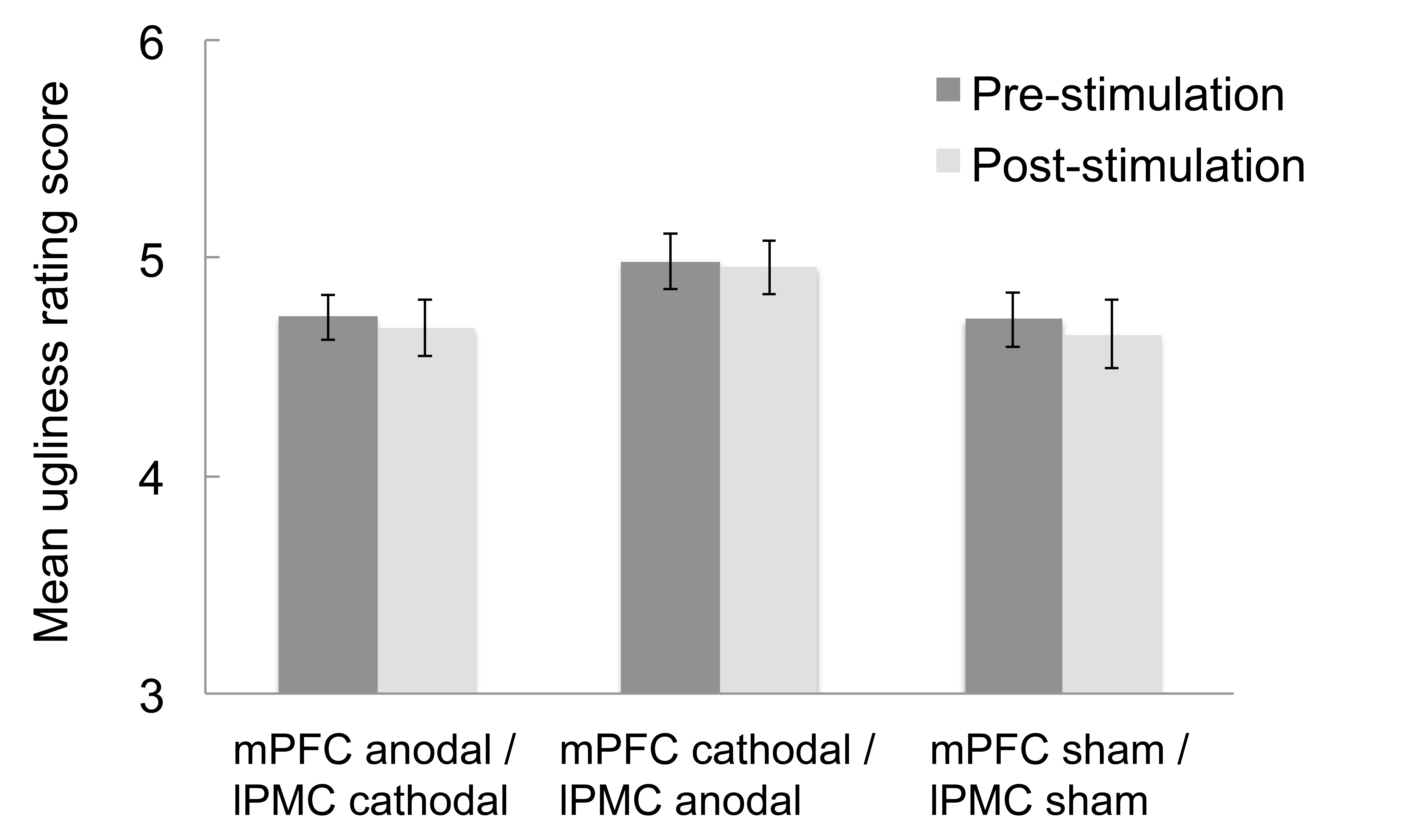


**Figure S3. Pre- and post-stimulation ugliness rating scores in different stimulation conditions.** Error bars represent SEs of the mean.

## Pre- and post-stimulation ugliness rating latencies in different stimulation conditions

Figure S4 shows the mean ugliness rating latencies in the pre- and post-stimulation sessions. A 3 (group: mPFC anodal vs. mPFC cathodal vs. sham) × 2 (session: pre-stimulation vs. post-stimulation) ANOVA on the mean ugliness rating latencies revealed a significant main effect of session (*F*(1, 42) = 7.49, *p* < .01, η_p_^2^ = .15), suggesting rating latencies in the post-session were faster than those in the pre-session. However, there were neither significant main effect of group (*F*(2, 42) = 0.57, *p* = .57, η_p_^2^ = .03) nor significant interaction effect between group and session (*F*(2, 42) = 1.31, *p* = .28, η_p_^2^ = .06), indicating that tDCS did not affect ugliness rating latency.


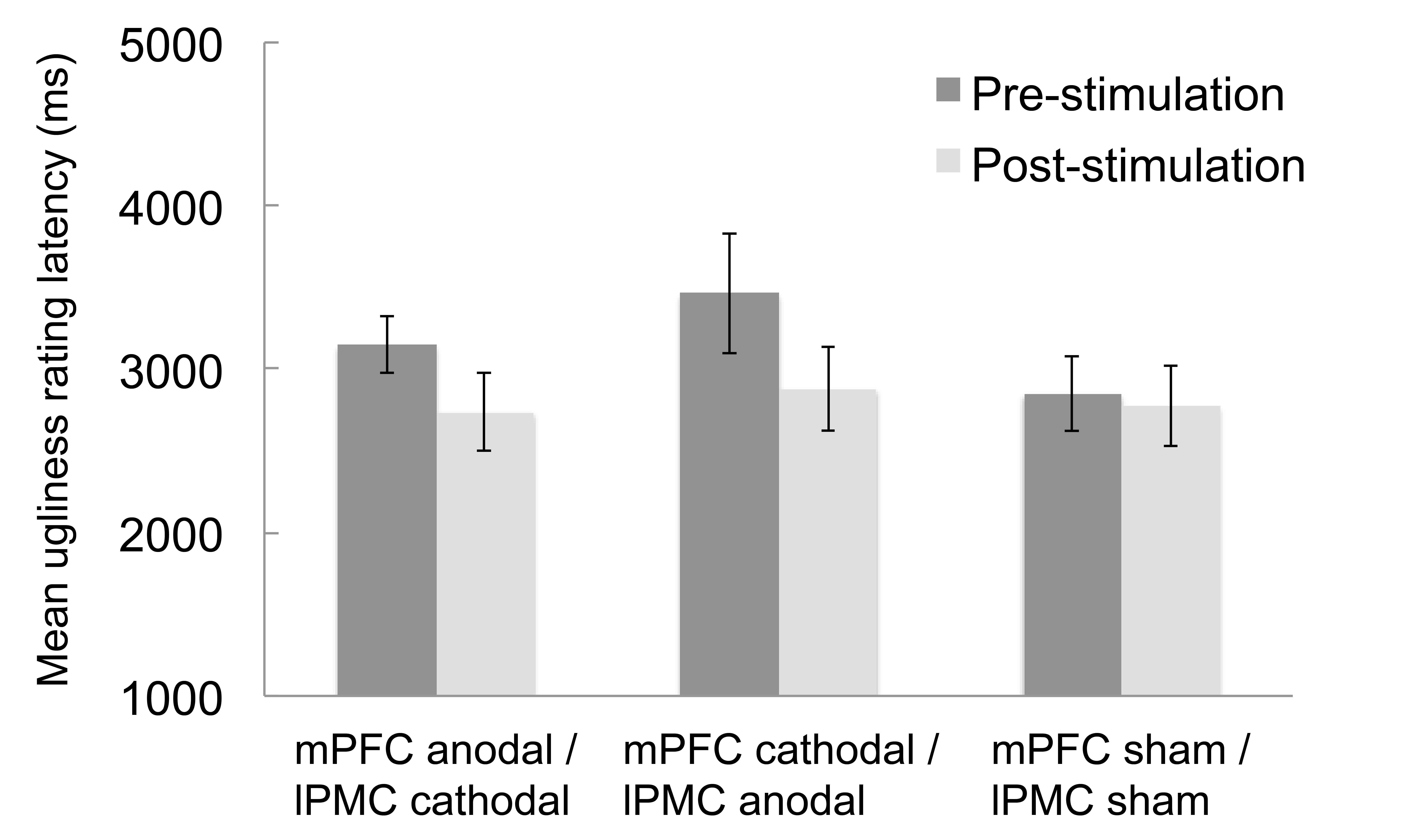


**Figure S4. Pre- and post-stimulation ugliness rating latencies in different stimulation conditions.** Error bars represent SEs of the mean.

## Response rate of judging “consistent” in the beauty-ugliness judgment task

Figure S5 shows the mean response rate of judging “consistent” in the beauty-ugliness judgment task. We conducted a 3 (group: mPFC anodal vs. mPFC cathodal vs. sham) × 2 (session: pre-stimulation vs. post-stimulation) × 2 (judgment: beauty vs. ugliness) ANOVA on the response rate that participants judged their aesthetic reaction was consistent with the meaning of the character (i.e., beauty or ugliness). The analysis revealed neither significant main effects (group: *F*(2, 42) = 0.67, *p* = .51, η_p_^2^ = .03; session: *F*(1, 42) = 0.70, *p* = .41, η_p_^2^ = .02; judgment: *F*(1, 42) = 0.49, *p* = .49, η_p_^2^ = .01), nor significant two-way interaction effects (group × session: *F*(2, 42) = 3.00, *p* = .06, η_p_^2^ = .13; group × judgment: *F*(2, 42) = 1.84, *p* = .17, η_p_^2^ = .08; session × judgment: *F*(1, 42) = 0.14, *p* = .72, η_p_^2^ = .00). There was no significant three-way interaction effect (*F*(2, 42) = 2.98, *p* = .06, η_p_^2^ = .12).





# Figure S5. Response rate of judging “consistent” in the beauty-ugliness judgment task. Error bars represent SEs of the mean.

## Individual scores of changes in ratings of beauty and ugliness evaluations

## Figure S6 shows the individual scores of changes in ratings of beauty and ugliness evaluations. Each colored circle in Figure S6 represents a single participant’s data observed in beauty and ugliness rating tasks.

##
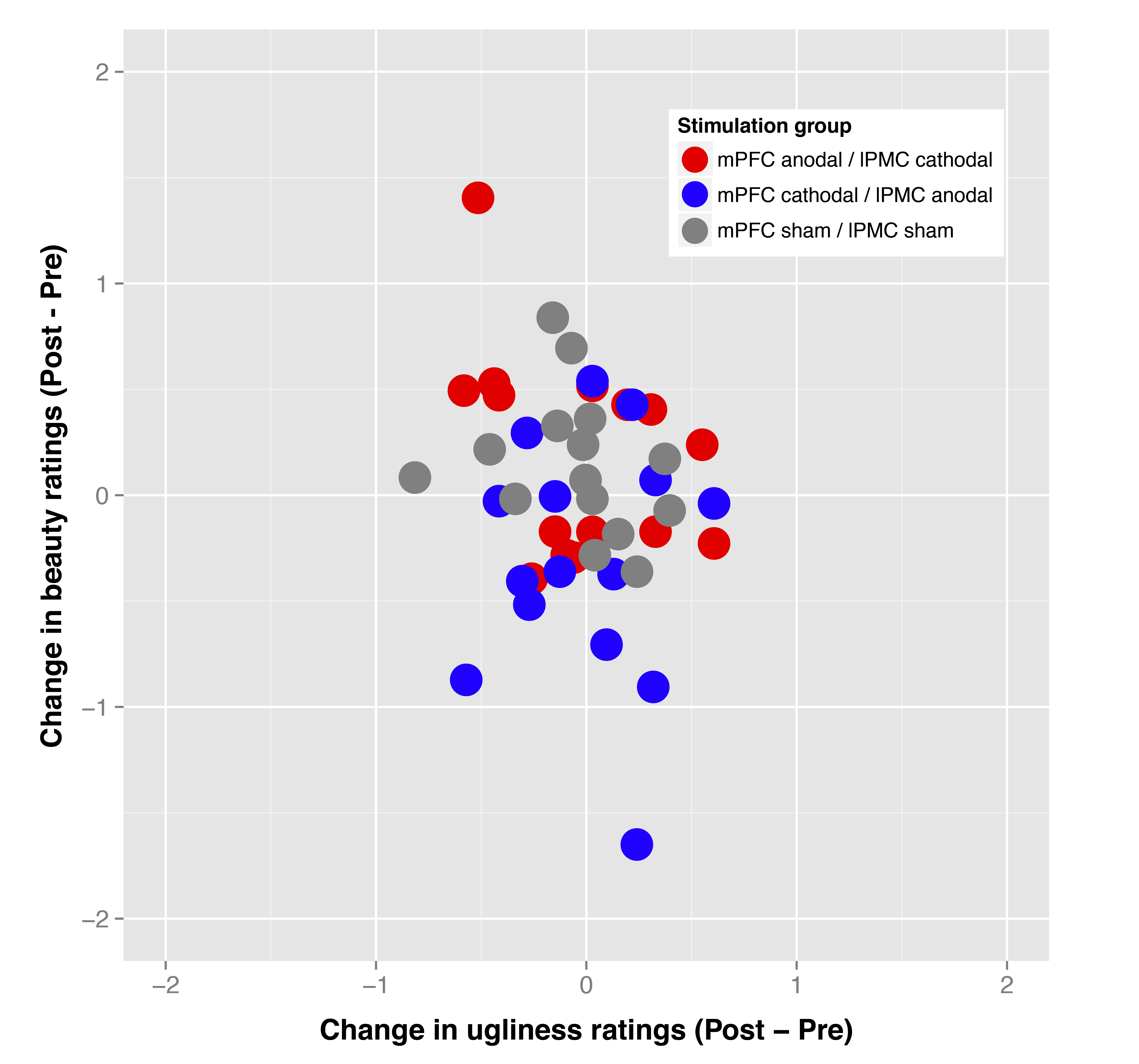


**Figure S6. Individual scores of changes in ratings of beauty and ugliness evaluations**.
